# The endocannabinoid system in bovine tissues: characterization of transcript abundance in the growing Holstein steer

**DOI:** 10.1186/s12917-024-04319-x

**Published:** 2024-10-22

**Authors:** Coral Kent-Dennis, James L. Klotz

**Affiliations:** USDA-ARS Forage-Animal Production Research Unit, University of Kentucky Campus, 1100 S. Limestone Rd. N220 Ag. Science North, Lexington, KY 40546 USA

**Keywords:** Endocannabinoids, Ruminant, Cattle

## Abstract

**Background:**

The endocannabinoid system (ECS) is highly integrated with seemingly all physiological and pathophysiological processes in the body. There is increasing interest in utilizing bioactive plant compounds, for promoting health and improving production in livestock. Given the established interaction between phytochemicals and the ECS, there are many opportunities for identification and development of therapies to address a range of diseases and disorders. However, the ECS has not been thoroughly characterized in cattle, especially in the gastrointestinal tract. The objective of this study was to characterize the distribution and transcriptional abundance of genes associated with the endocannabinoid system in bovine tissues.

**Methods:**

Tissues including brain, spleen, thyroid, lung, liver, kidney, mesenteric vein, tongue, sublingual mucosa, rumen, omasum, duodenum, jejunum, ileum and colon were collected from 10-mo old Holstein steers (*n* = 6). Total RNA was extracted and gene expression was measured using absolute quantification real time qPCR. Gene expression of endocannabinoid receptors *CNR1* and *CNR2*, synthesis enzymes *DAGLA*, *DAGLB* and *NAPEPLD*, degradation enzymes *MGLL* and *FAAH*, and transient receptor potential vanilloids *TRPV3* and *TRPV6* was measured. Data were analyzed in R using a Kruskal-Wallis followed by a Wilcoxon rank-sum test. Results are reported as the median copy number/20 ng of equivalent cDNA (CN) with interquartile range (IQR).

**Results:**

The greatest expression of *CNR1* and *CNR2* was in the brain and spleen, respectively. Expression of either receptor was not detected in any gastrointestinal tissues, however there was a tendency (*P* = 0.095) for *CNR2* to be expressed above background in rumen. Expression of endocannabinoid synthesis and degradation enzymes varied greatly across tissues. Brain tissue had the greatest *DAGLA* expression at 641 CN (IQR 52; *P* ≤ 0.05). *DAGLB* was detected in all tissues, with brain and spleen having the greatest expression (*P* ≤ 0.05). Expression of *NAPEPLD* in the gastrointestinal tract was lowest in tongue and sublingual mucosal. There was no difference in expression of *NAPEPLD* between hindgut tissues, however these tissues collectively had 592% greater expression than rumen and omasum (*P* ≤ 0.05). While *MGLL* was found to be expressed in all tissues, expression of *FAAH* was only above the limit of detection in brain, liver, kidney, jejunum and ileum. *TRPV3* was expressed above background in tongue, rumen, omasum and colon. Although not different from each other, thyroid and duodenum had the greatest expression of *TRPV6*, with 285 (IQR 164) and 563 (IQR 467) CN compared to all other tissues (*P* < 0.05).

**Conclusions:**

These data demonstrate the complex distribution and variation of the ECS in bovine tissues. Expression patterns suggest that regulatory functions of this system are tissue dependent, providing initial insight into potential target tissues for manipulation of the ECS.

**Supplementary Information:**

The online version contains supplementary material available at 10.1186/s12917-024-04319-x.

## Introduction

**Fig. 1:**
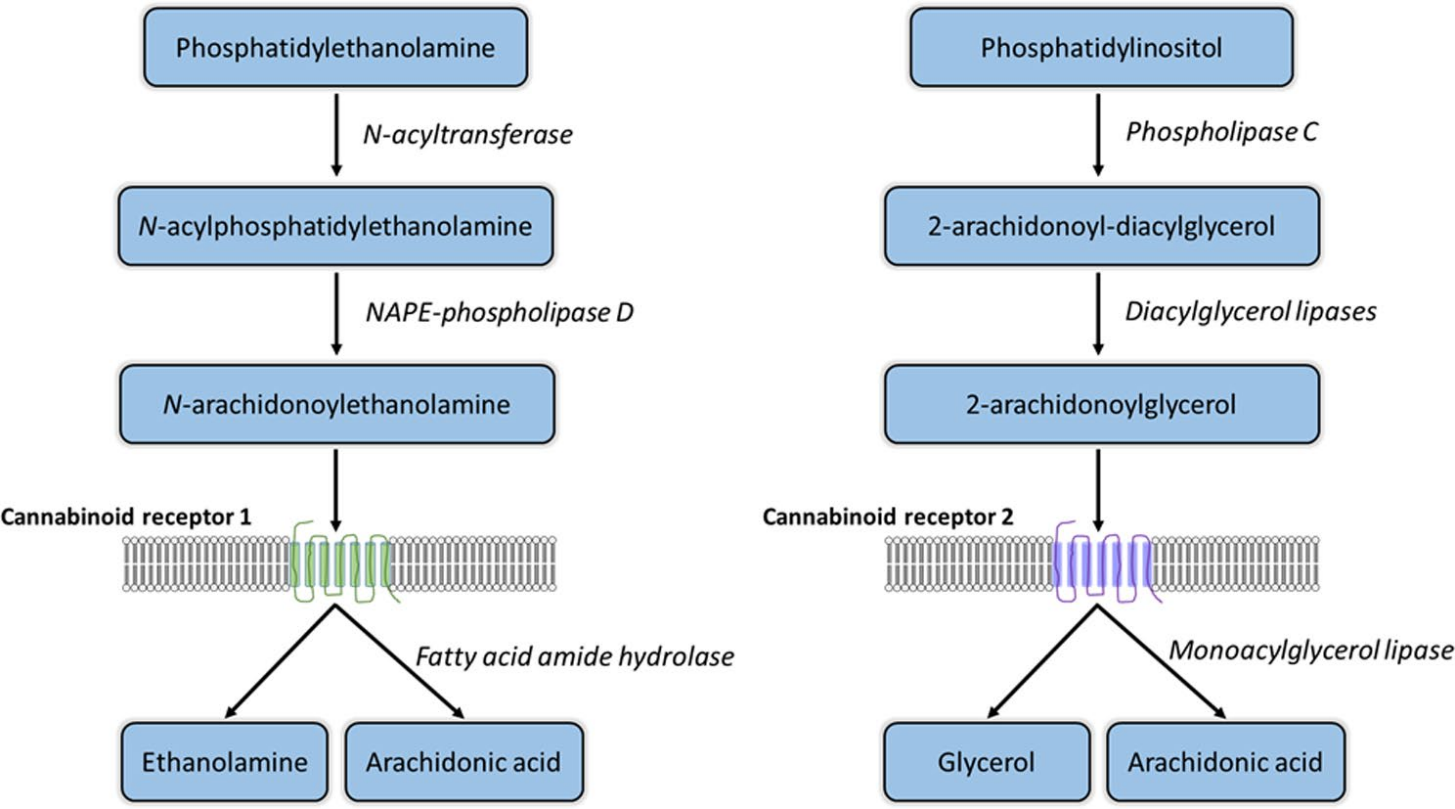
Simplified, canonical biosynthesis and degradation pathways for endocannabinoids N-arachidonoylethanolamine (AEA) and 2-arachidonoylglycerol (2-AG), derived from the membrane phospholipid precursors phosphatidylethanolamine and phosphatidylinositol, respectively. Alternative synthesis pathways are not shown

The endocannabinoid system (ECS) is a complex cell-signaling mechanism active in all vertebrate species. The ECS utilizes lipid mediated signaling through the actions of several endogenous cannabinoid molecules including N-arachidonoylethanolamine (AEA) and 2-Arachidonoylglycerol (2-AG), which act on two canonical, G protein-coupled receptors, cannabinoid receptor 1 (CB1) and cannabinoid receptor 2 (CB2), which are encoded by the *CNR1* and *CNR2* genes, respectively. Additional components of the ECS include a number of enzymes responsible for cellular synthesis and degradation (Fig. 1) of endocannabinoids [1]. Since the initial discovery of the CB1 receptor in tissue of the central nervous system [2], the various components of the ECS have been identified, at various levels of expression and localization, in a wide variety of human and rodent tissues [3]. The ECS is highly interconnected with other systems, playing a role in seemingly most physiological and pathophysiological processes [4].

Initially research in this area was heavily focused on elucidating the psychotropic effects of phytocannabinoids, and in particular delta-9-tetrahydrocannabinol (THC) derived from *Cannabis sativa*. However, more recent work has identified many plant-derived bioactive compounds capable of interacting with the ECS and producing potentially beneficial effects [4]. For example, both THC and cannabidiol (CBD) have been associated with reduced intestinal inflammation and motility in a model of colitis in rats [5]. Compounds such as yangonine, found in kava (*Piper methysticum*), have been shown to interact with both CB1 and CB2 [6]. β-caryophyllene (BCP), found in essential oils from a variety of plants including cloves and hops [7], is a full CB2 agonist and has shown promise as a potential therapy for multiple diseases including colitis [8] and diabetes [9]. In addition, other components of the ECS, such as the degradation enzyme fatty acid amide hydrolase (FAAH), or various transient receptor potential (TRP) channels continue to be investigated for their potential role in pain and inflammation [10, 11].

The wide distribution throughout the tissues in the body observed in humans and rodents, and the pleiotropic nature of the ECS suggest potential for modulation of multiple physiological systems. Given the well-established interaction between the ECS system and a range of plant-derived bioactive compounds, there are ample opportunities for development of therapies to address a range of diseases and disorders [4]. However, work to date has predominantly been in human tissues or cells, or in model species with an emphasis on rodents [3]. In contrast, relatively few studies have investigated the distribution and functionality of the ECS in ruminants, despite several recent studies demonstrating the potential utility of phytocannabinoids in dairy cows. Of particular interest are data suggesting regulatory effects of the ECS on lipid metabolism [12, 13] and dry matter intake regulation [14, 15]. Identifying novel physiological mechanisms that directly impact production and health is imperative for improving efficiency and sustainability of food animals.

There is a particular void, however, of information on the ECS and the gastrointestinal tract in ruminants, where there may be many opportunities for use of bioactive compounds in order to improve health, intake or metabolism, or as alternatives to antibiotics [16]. Moreover, while the components of the ECS have been well characterized in rodents and humans, evidence suggests that the level of expression and the specific localization varies substantially with species [3]. Therefore, the primary objective of this study was to characterize the expression of key genes associated with the steady state ECS in an extensive distribution of bovine gastrointestinal and internal tissues. It was expected that components of the ECS would be expressed in the small intestinal and internal organ tissues at various levels, however the expression in foregut and oral tissues remains to be elucidated. A wide range of tissues were selected in order to provide a comprehensive characterization. The overall aim of this study was to provide a starting point for exploring novel targets for the bovine ECS.

## Materials and methods

### Animals and tissue collection

All procedures that required the use of animals were approved prior to initiation of work by the University of Kentucky Animal Care and Use Committee (2020–3479). Holstein steers (*n* = 6) were fed a common, alfalfa-based diet [17] at the University of Kentucky Beef Unit and slaughtered between 10 and 12 months of age at approximately 250 kg body weight. Steers were stunned by penetrating captive bolt and subsequently exsanguinated in the University of Kentucky Meats Laboratory. All tissues for the present experiment were collected post-slaughter within 30 min of death. A total of 15 tissues collected for evaluation of gene expression included brain (BRN), tongue (TNG), sublingual mucosa (SLM), rumen (RUM), omasum (OMA), duodenum (DUO), mid-jejunum (MJE), ileum (ILE), colon (COL), thyroid (THY), lung (LNG), liver (LVR), kidney (KID), spleen (SPN) and mesenteric vein (MVN). Each tissue was collected from a consistent anatomical location. Sample of the BRN were obtained by removal of the frontal bone and frontal lobe with a saw and the anterior lobe of the cerebellum was subsequently excised. Epithelium of the TNG was collected from the right-lateral side, mid-body, and stripped of underlying muscle tissues. The SLM was taken from the floor of the mouth, avoiding ducts and glandular tissue. Samples of the rumen consisted of papillae (~ 20) from craniodorsal sac were clipped at the base and rinsed in PBS. The OMA mucosa was collected from a central region of the laminae was rinsed in PBS to remove digesta. Whole segments of the DUO, MJE (~ 1 cm^2^) were collected approximately 1 and 10 m caudal to the pyloric sphincter respectively. Similar segment of the ILE and COL were taken approximately 9 m cranial and 1 m caudal to the ileocecal junction, respectively. THY was isolated from the dorsal surface of the trachea immediately caudal to the larynx, the associated fat and connective tissues were removed and a subsample isolated from the left lobe. Sample of the LNG and LVR were taken from the apical tip of the right and left lobes of the respective organs. A combined sample of renal cortex and medulla were isolated from either left of right KID. Samples of the SPN including the capsule and red/white pulp were taken from the tip of ventral end. Finally, a ~ 1 cm in length of the MVN was isolated from the collateral branch proximal to the ileal fold, as previously described [18]. Tissues were immediately snap frozen in liquid nitrogen and subsequently stored at -80. Additional replicate samples from the gastrointestinal tract were fixed in 10% neutral buffered formalin for 48-h prior to processing and paraffin embedding.

### Gene expression analysis

Frozen tissue samples were ground with a mortar and pestle under liquid nitrogen. Approximately 100 mg of ground tissue was used to extract total RNA with a phenol-chloroform isolation procedure [19] with double isopropanol precipitations. The total RNA was quantified with a spectrophotometer (Nanodrop One; Thermo Fisher Scientific, Waltham, MA, USA), and samples were treated with DNase (Turbo DNase kit; Thermo). RNA integrity was determined using a bioanalyzer (2100; Agilent, Santa Clara, CA, USA) and all samples were confirmed to have RNA integrity numbers (RIN) of at least 7. Two micrograms of total RNA from each sample was reverse transcribed (High Capacity cDNA Reverse Transcription Kit; Thermo) and subsequently diluted to a final concentration of 10 ng/µl cDNA assuming 100% conversion. Primer sequences for genes of interest (Table 1) were designed using the current RefSeq mRNA sequences and spanned exon-exon junctions as identified using the BLAST-like Alignment Tool (BLAT), relative to the *Bos taurus* assembly ARS-UCD2.0. Melt curve analysis was used to verify production of a single amplicon and efficiencies between 90 and 105% established using a serial dilution of pooled cDNA. The target amplicon product was produced using each validated primer pair in cDNA from tissues with established expression, and the resulting product inserted into the pCR4 vector (TOPO TA Cloning Kit; Thermo). Circularized vector was then chemically transfected into TOP10 *Escherichia coli*, and colony selection carried out on Kanamycin selection plates. Selected colonies were expanded in LB broth, plasmids purified (GeneJet Plasmid Miniprep Kit, manufacturer info) and insertion verified by qPCR and restriction digest. Linearized plasmid was used to generate a 6-point standard curve from 10^7^ to 10^2^ copies/µl, as previously described by Ison et al. [19]. Efficiency of the standard curves were again verified between 90 and 105% and subsequently used for absolute quantification qPCR of gene expression, in order to compare expression across tissues. All qPCR was performed in duplicate with 20 ng cDNA, with Fast SYBR Green Master Mix (Thermo) in a real-time PCR system (StepOnePlus; Thermo).

**Table 1:** Gene-specific primer sequences

Gene Name (official symbol)	Gene ID	Target RefSeq^1^	Sequence (5’ – 3’)^2^	Annealing Temperature (ºC)	Amplicon Length (bp)
Cannabinoid receptor 1 (*CNR1*)	100,299,449	NM_001242341.2	F: AGGTTCGGGCTAAGGAAGAG R: GCACCACTGAGGATGAAGTG	64	114
Cannabinoid receptor 2 (*CNR2*)	539,769	NM_001192303.1	F: ATGAGGGACCTGGAGGAGAT R: AACAGGAAGAAGGGCTGTCC	62	93
NAPE^3^-phospholipase D (*NAPEPLD*)	541,291	NM_001015680.1	F: GATCACAGCAGCGTTCCAT R: TCCAGCTTCTTCAGGGTCAT	60	90
diacylglycerol lipase alpha (*DAGLA*)	523,665	NM_001192583.3	F: AGGAGGAGCCCACGTACTTC R: TTCTCCAGCACCTTGTTGAG	60	130
diacylglycerol lipase beta (*DAGLB*)	538,021	NM_001083487.1	F: AGCTACCTGATCGTGCTCCT R: ACAGATGGTCCCTTTCATGC	60	90
Fatty acid amide hydrolase (*FAAH*)	540,007	NM_001099102.2	F: GGAGGGTGACTGTGTGGTG R: CAAAGCTGAACATGGACTGG	60	92
Monoglyceride lipase (*MGLL*)	505,290	NM_001206681.1	F: CGAGGAATAAGACGGAGGTG R: GAAGGGCAGTGTCAGCTTG	67	140
Transient receptor potential vanilloid 3 (*TRPV3*)	523,538	NM_001099024.1	F: GACGTGCCTGACTTCCTCAT R: CTTGGTGTTGGGGTTGATGT	60	96
Transient receptor potential vanilloid 6 (*TRPV6*)	614,878	NM_001206189.1	F: GGCAACACTGTGATGTTCCA R: GAGGAGTCGATTTCCGTGAG	60	107

^1^National Center for Biotechnology Information (NCBI)

^2^F = forward; R = reverse

^3^NAPE = N-acyl phosphatidylethanolamine

### Immunohistofluorescence

Targets for immunohistofluorescence, CB2 and DAGLB, were selected *post hoc*. Samples of TNG, RUM, DUO, ILE and COL fixed in neutral buffered formalin (10% formaldehyde in phosphate buffer, Sigma-Aldrich, St. Louis, MO, USA) were processed, embedded and sectioned at the Pathology Research Core at the University of Kentucky. Four-micron sections were mounted onto glass slides, baked for 20 min at 60ºC and deparaffinized in xylene, followed by rehydration in decreasing concentrations of ethanol. Samples were subject to heat-induced antigen retrieval for 30 min at 90ºC with Tris-EDTA buffer (10 mM Tris, 1 mM EDTA, 0.05% Tween20, pH 9.0) for TNG, DUO, ILE and COL or citrate buffer (10 mM citric acid, 2 mM EDTA, 0.05% Tween 20, pH 6.2) for RUM. Following antigen retrieval, sections were blocked with 100% FBS for 2 h. Primary antibodies including rabbit anti-CB2 (Final concentration 0.01 mg/mL; Novus Biologicals, LLC, Littleton, CO, USA) and rabbit anti-DAGLB (Final concentration 0.005 mg/mL; Abcam, Cambridge, UK) were diluted with incubation buffer (1% BSA 1% horse serum and 0.01% sodium azide in PBS). Sections were incubated with primary antibody or isotype control (ChromPur Rabbit IgG, Jackson ImmunoResearch Inc, West Grove, PA, USA) overnight at 4ºC. Slides were subsequently washed 3 times in PBS with Tween (0.05% Tween20, pH 7.4; PBS-T) and incubated with secondary antibody (Anti-Rabbit AlexaFluor488, Abcam), diluted to a final concentration of 0.004 mg/mL 1:500 with incubation buffer, for 2 h at room temperature. Slides were again washed 3 times and then counterstained with 4′,6-diamidino-2-phenylindole (DAPI), diluted to 300 nM in PBS. After washing once more, slides were coverslipped with Mowiol mounting medium (made in-house, all ingredients sourced from Sigma) and imaged using an Axiovert 200 fluorescent microscope (Zeiss, Oberkochen, Germany). Non-specific staining was evaluated in all tissues using a rabbit isotype control, and minimal interaction with nonspecific rabbit antibodies was observed (Additional File 1). All staining procedures and imaging were conducted under the same conditions as the target primary antibodies.

### Statistical analysis

Statistical analysis was performed using R v4.3.2 [20]. Data were analyzed using a Kruskal-Wallis followed by a Wilcoxon rank-sum test and Benjamini-Hochberg used for multiple comparisons. Gene expression data were normalized and presented as median transcript copy number (CN) per 20 ng of equivalent cDNA [21], with interquartile range (IQR). A threshold of 10 CN/20 ng cDNA was used as the cutoff for the level of detection. Data visualization was carried out using the ggplot2 package [22]. Differences were considered significant at *P* < 0.05.

## Results

**Fig. 2:**
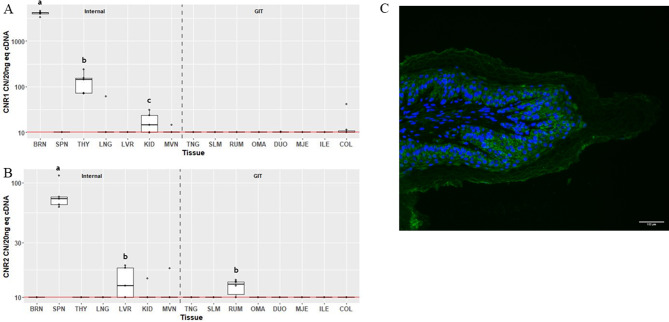
Gene expression of cannabinoid receptors *CNR1* (**A**), *CNR2* (**B**) in internal organ and gastrointestinal tissues (GIT), and localization of CB2 (green) in ruminal papillae, counterstained with DAPI (**C**). Data are presented as median transcript copy number (CN) per 20 ng of equivalent cDNA. *Abbreviations* BRN = brain, SPN = spleen, THY = thyroid, LNG = lung, LVR = liver, KID = kidney, MVN = mesenteric vein, TNG = tongue, SLM = sublingual mucosa, RUM = rumen, OMA = omasum, DUO = duodenum, MJE = mid-jejunum, ILE = ileum, COL = colon. Unique letters between tissues indicate significance (*P* < 0.05)

The greatest expression of *CNR1* (Fig. 2A) was observed in the BRN, followed by the THY, at 4106 (IQR 299) and 144 (IQR 81.5) CN/20 ng cDNA, respectively (*P* = 0.035). *CNR1* expression above 10 CN/20 ng cDNA in KID was detected in 3 of the samples, however median expression was not significantly different than background. Expression of *CNR1* was not detectable in the other 12 tissues. Expression of *CNR2* (Fig. 2B) was only detectable above background in SPN at 72 CN/20 ng cDNA (IQR 11.1; *P* = 0.043). Median expression in LVR tended to be above background (*P* = 0.104). There was also a tendency (*P* = 0.095) for *CNR2* to be expressed above background in RUM. Localization of CB2 in RUM was detected as cytosolic staining in the epithelial cells, especially in the *stratum basale*,* spinosum* and *granulosum* (Fig. 2C). CB2 was largely absent from the *stratum corneum*.

**Fig. 3:**
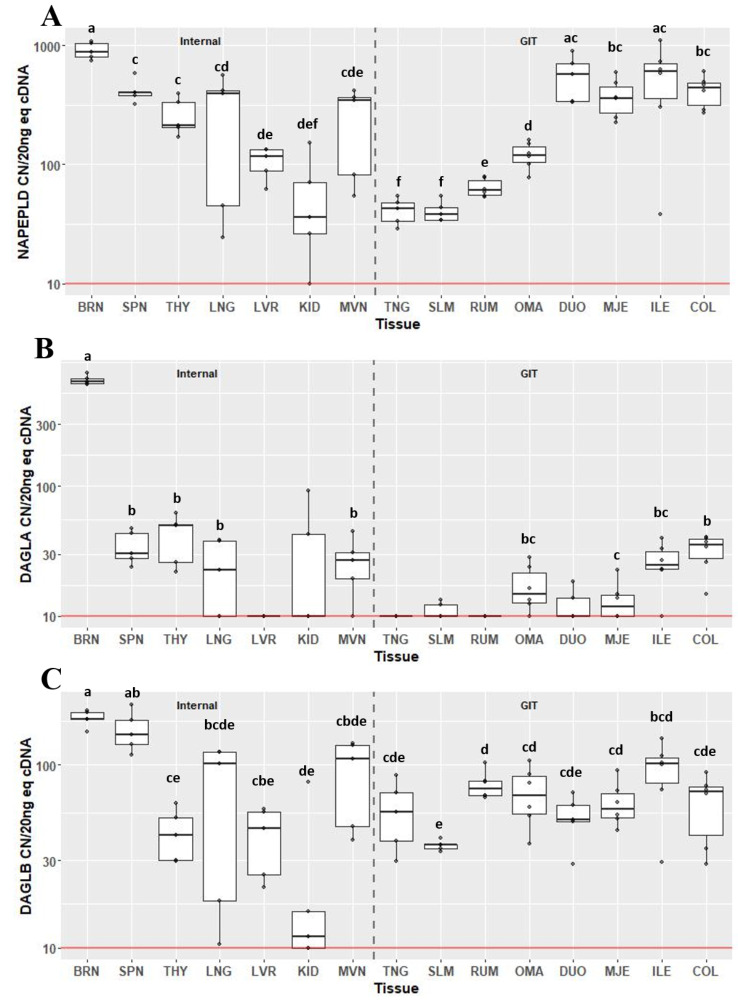
Gene expression of endocannabinoid synthesis enzymes *NAPEPLD* (**A**), *DAGLA* (**B**) and *DAGLB* (**C**) in internal organ and gastrointestinal tissues (GIT). Data are presented as median transcript copy number (CN) per 20 ng of equivalent cDNA. *Abbreviations* BRN = brain, SPN = spleen, THY = thyroid, LNG = lung, LVR = liver, KID = kidney, MVN = mesenteric vein, TNG = tongue, SLM = sublingual mucosa, RUM = rumen, OMA = omasum, DUO = duodenum, MJE = mid-jejunum, ILE = ileum, COL = colon. Unique letters between tissues indicate significance (*P* < 0.05)

Expression of the endocannabinoid synthesis enzymes, *NAPEPLD* (Fig. 3A), *DAGLA* (Fig. 3B) and *DAGLB* (Fig. 3C), varied greatly across tissues. Expression of *NAPEPLD* was detectable above background in all 15 tissue types examined. With the exception of the DUO and ILE, the BRN had the greatest expression of *NAPEPLD* compared to other tissues (*P* < 0.05) at 880 CN/20 ng cDNA (IQR 248). Within the gastrointestinal tract, the tissues of the small intestine (DUO, MJE and ILE) and COL had greater expression (collectively greater than 300 CN) of *NAPEPLD* compared with tissues of the foregut and the mouth. Expression was greater in OMA compared to RUM (CN 119, IQR 37.9 vs. CN 60.7, IQR 18.4; *P* = 0.028). Both TNG and SLM had the lowest expression compared to all other GIT tissues (CN 43, IQR 14.7 and CN 38, IQR 8.8), however they were not different from each other. The greatest expression of *DAGLA* was in the BRN at 641 CN/20 ng cDNA (IQR 52; *P* < 0.05) followed by THY (CN 49.5, IQR 24.6) and SPN (CN 30.3, IQR 15.5), which were not different from each other (Fig. 2B). Although expression was detected above background in two samples of KID, median CN of *DAGLA* was not above background. Within the gastrointestinal tract, expression of *DAGLA* in COL was greater compared to MJE (*P* < 0.05), and there was a tendency for COL to have greater expression than OMA (*P* = 0.073). COL and ILE did not significantly differ. Median expression of *DAGLA* in TNG, SLM, RUM and DUO did not differ from background. All tissues were found to express *DAGLB* above background, with the greatest expression in BRN and SPN (*P* < 0.05), which were not different from each other. Except for LNG, MVN and ILE, expression of *DAGLB* in SPN was greater than other tissues (*P* < 0.05). Within the gastrointestinal tract, expression of *DAGLB* was least in SLM at 36.4 CN (IQR 1.9) compared to RUM (CN 74.1, IQR 13.9; *P* = 0.047) and MJE (CN 57.6, IQR 18.2; *P* = 0.047).

**Fig. 4:**
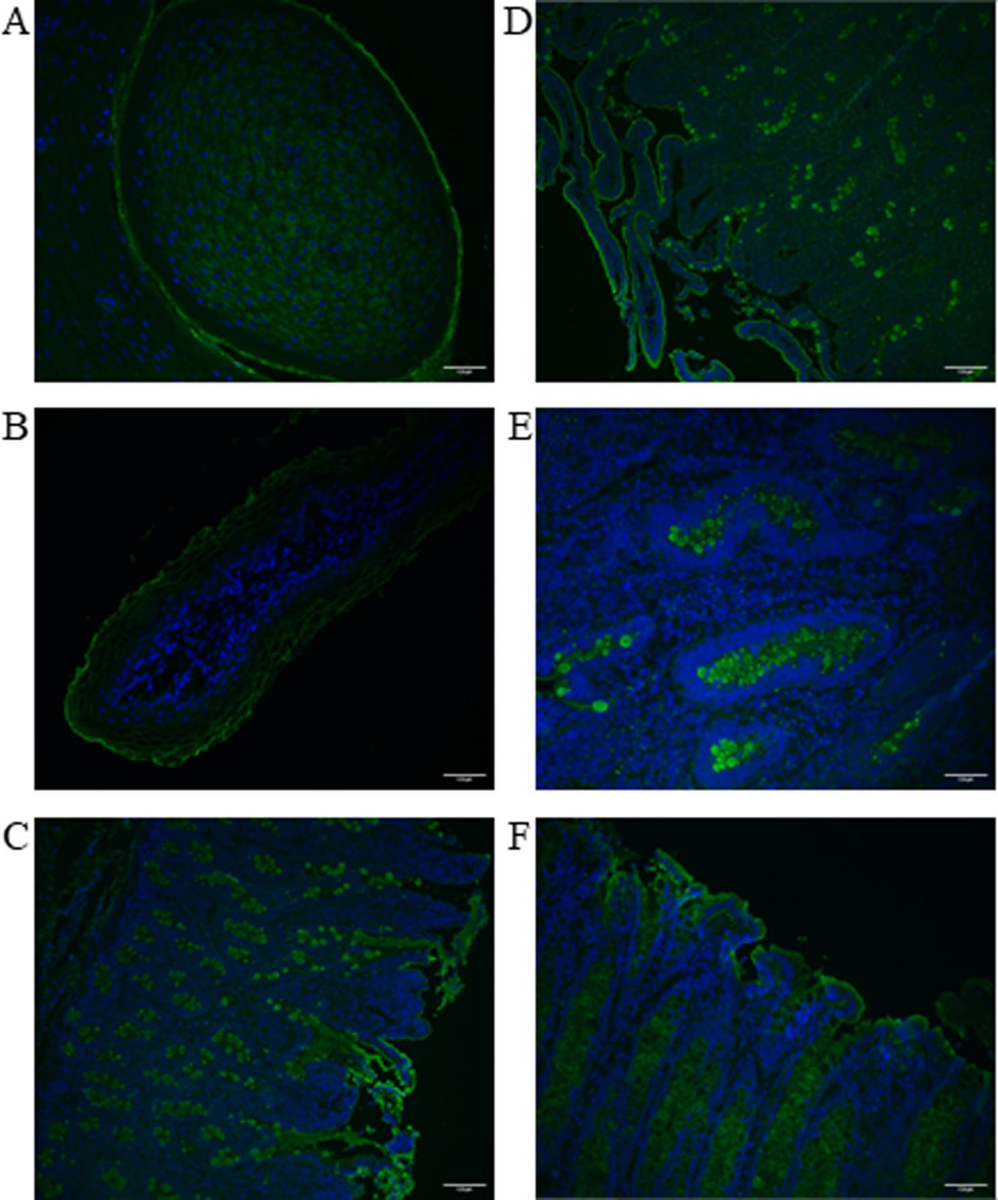
Localization of DAGLB (green) in circumvallate papillae of the tongue (**A**), ruminal papillae (**B**), epithelium of duodenum (**C**), ileum at 10X (**D**) and ileum at 40X (**E**), and in colon epithelium (**F**). Nuclei are counterstained with DAPI (blue)

The circumvallate papillae of the tongue showed positive, cytoplasmic staining for DAGLB (Fig. 4A). Staining of DAGLB in the RUM was localized to the plasma membrane (Fig. 4B). In both the duodenum (Fig. 4C) and ileum (Fig. 4D and E), there was strong positive staining for DAGLB in goblet cells, but staining was not observed within the enterocytes. In colon, DAGLB appeared to be localized to either goblet cells or enterocytes, and was dependent on animal (Fig. 4F).

**Fig. 5:**
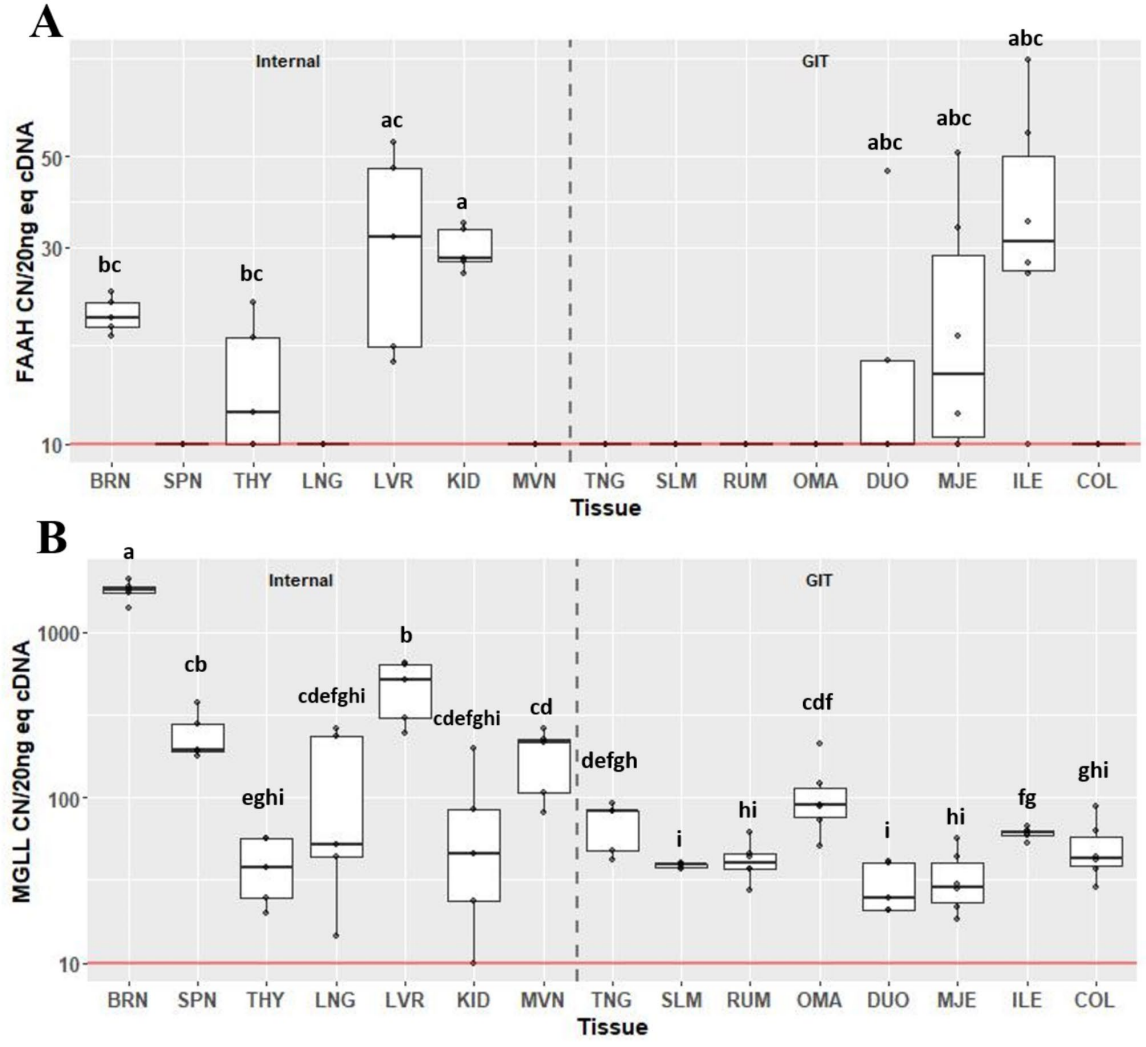
Gene expression of endocannabinoid degradation enzymes *FAAH* (**A**) and *MGLL* (**B**) in internal organ and gastrointestinal tissues (GIT). Data are presented as median transcript copy number (CN) per 20 ng of equivalent cDNA. *Abbreviations* BRN = brain, SPN = spleen, THY = thyroid, LNG = lung, LVR = liver, KID = kidney, MVN = mesenteric vein, TNG = tongue, SLM = sublingual mucosa, RUM = rumen, OMA = omasum, DUO = duodenum, MJE = mid-jejunum, ILE = ileum, COL = colon. Unique letters between tissues indicate significance (*P* < 0.05)

Expression of degradation enzymes *FAAH* (Fig. 5A) and *MGLL* (Fig. 5B) also varied considerably across tissues. Expression of *FAAH* was detected above background in six of the tissues assessed, including BRN, THY, LVR, KID, MJE and ILE. Expression of *FAAH* in KID was greater at 28.3 CN (IQR 5.61) compared to BRN (CN 20.3, IQR 2.85; *P* = 0.032) and THY (CN 11.9, IQR 8.15; *P* = 0.032). Expression of *MGLL* was detectable above background in all tissues. Within internal organs, expression was greatest in BRN at 1798 CN (IQR 160). Expression of *MGLL* in SPN was greater compared to THY (CN 194, IQR 89.4 vs. CN 37.5, IQR 32.4; *P* = 0.027). Although not different from SPN, expression in LVR was greater compared to THY (CN 515, IQR 333 vs. CN 37.5, IQR 32.4; *P* = 0.027), and was also greater than MVN (CN 214, IQR 118; *P* = 0.044). Within gastrointestinal tissues, expression of *MGLL* was greatest in OMA, at 87.5 CN (IQR 37), compared to SLM, RUM, DUO, MJE and COL (*P* < 0.05). Expression did not differ between OMA and TNG or ILE. Expression of *MGLL* in ILE was greatest, at 61.3 CN (IQR 4.0), compared to SLM, RUM, DUO and MJE (*P* < 0.05).

**Fig. 6:**
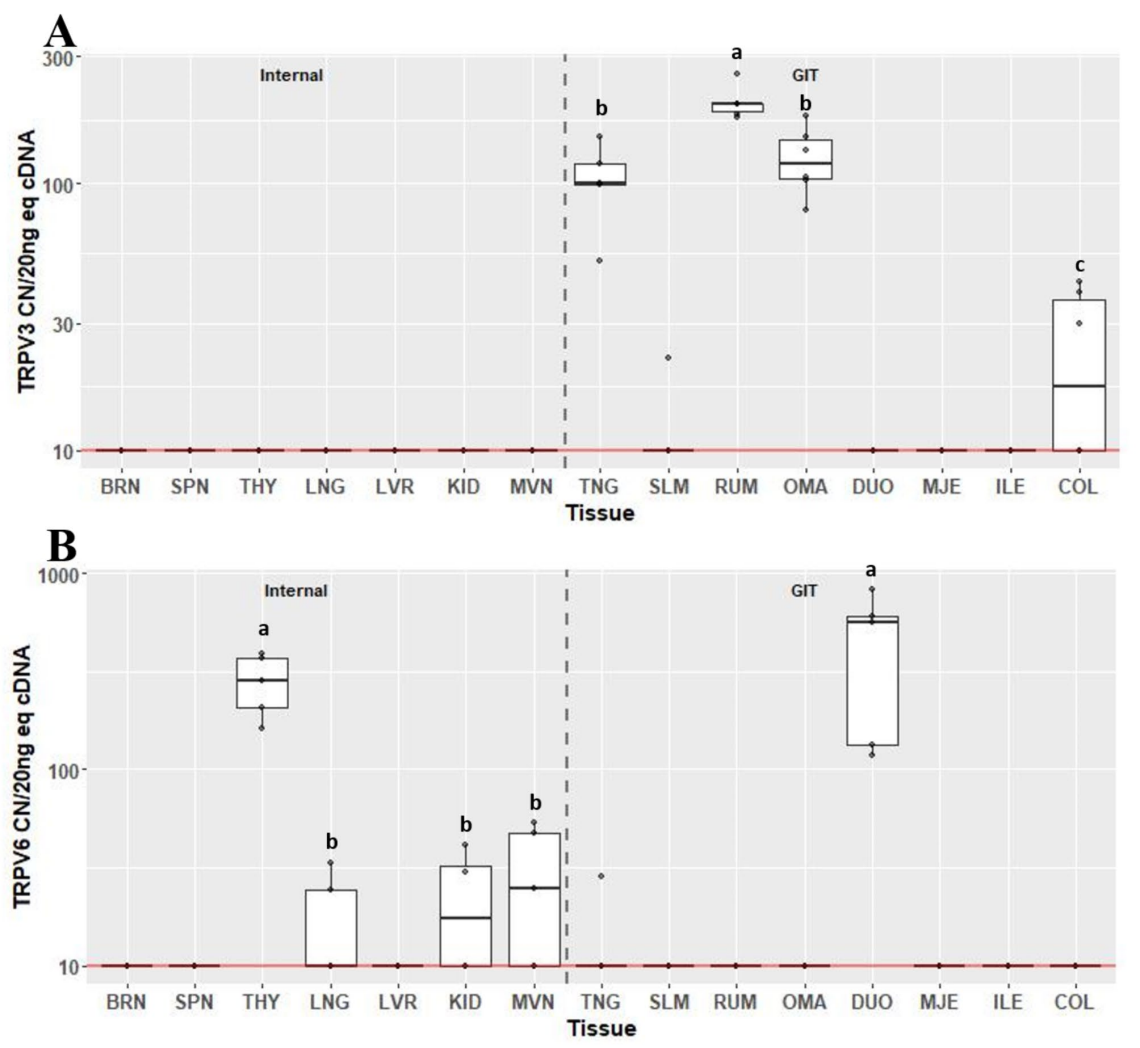
Gene expression of transient receptor potential vanilloids *TRPV3* (**A**) and *TRPV6* (**B**) in internal organ and gastrointestinal tissues (GIT). Data are presented as median transcript copy number (CN) per 20 ng of equivalent cDNA. *Abbreviations* BRN = brain, SPN = spleen, THY = thyroid, LNG = lung, LVR = liver, KID = kidney, MVN = mesenteric vein, TNG = tongue, SLM = sublingual mucosa, RUM = rumen, OMA = omasum, DUO = duodenum, MJE = mid-jejunum, ILE = ileum, COL = colon. Unique letters between tissues indicate significance (*P* < 0.05)

Transient receptor potential vanilloids *TRPV3* (Fig. 6A) and *TRPV6* (Fig. 6B) were only detectable in 4/15 and 5/15 tissues respectively. Expression of *TRPV3* was not detected in any internal organ tissue, and was only found to be expressed in TNG, RUM, OMA and COL. Expression of *TRPV3* was greatest in RUM at 198 CN (IQR 13.3) and least in COL at 20 CN (IQR 27; *P* < 0.05). Expression of *TRPV6* was only detected above background in THY, KID, MVN and DUO. Expression was greatest in THY and DUO (CN 284, IQR 164 and CN 563, IQR 467, respectively; *P* < 0.05), but were not different from each other. Compared to THY, expression of *TRPV6* was less in KID and MVN (CN 20, IQR 22.8 and CN 24.6, IQR 37.4, respectively; *P* < 0.05), but were not significantly different from one another.

## Discussion

While a relatively new system in terms of research, especially in animals [3], increasing evidence suggests a multitude of opportunities for unlocking new therapies for diseases and disorders through manipulation of the ECS. Although the ECS is widely thought to be ubiquitously expressed throughout the body [23], it is increasingly recognized that distribution and physiological roles are cell or tissue-specific [23, 24]. Moreover, crucial differences in the ECS between species have been identified [3]. Therefore, a key step in furthering the knowledge base is an understanding of the steady state transcriptional abundance. The present study focused on elucidating the constitutive expression of the bovine ECS across a total of fifteen tissues, several of which (e.g. TNG, SLM, RUM, OMA), have not been investigated previously.

The two main receptors of the ECS, CB1 and CB2, are most commonly reported to be found in the BRN and various lymphoid tissues, respectively [25, 26]. The CB1 receptor has been detected throughout the different regions of the central nervous system in all mammals and most non-mammalian species [3, 27] and, in fact, is the most abundantly expressed receptor in the BRN [25]. Unsurprisingly, the *CNR1* gene was found to be highly expressed in the bovine cerebellum tissue examined as part of the current study. These results are consistent with findings from humans and rodents showing substantial expression of this receptor in cerebellum as well as neocortex and hippocampus [28, 29]. Interactions between CB1 receptors in the cerebellum and THC have been linked to altered cerebellar functions, such as cognition and motor control [30]. The expression levels in the present study suggest that phytocannabinoids may similarly alter the cerebellar functions in cattle. Indeed, Wagner et al. [31] demonstrated that dairy cows fed silage made with *Cannabis sativa* had significant motor impairment.

Outside the central nervous system, expression of the CB1 receptor has been found on numerous non-neuronal tissues [23]. In the current study, expression of *CNR1* was notable in the thyroid. Although there is a paucity of information about the role of the ECS in the thyroid gland, CB1 expression was found to be greater in patients with malignant compared to benign thyroid tumors [32]. In adult rats, not only was *CNR1* mRNA found expressed in the thyroid, but serum T3 and T4 levels were reduced when a CB1 agonist was administered [33]. Moreover, it has been reported that thyroid hormone levels can be influenced by the use of cannabis [34]. A growing body of evidence suggests allostatic modulation of the hypothalamic pituitary thyroid axis, termed non-thyroidal illness syndrome (NTIS) occurs in response to pathogenic infections in both humans and animals [35–37]. The ECS is known to be activated during an inflammatory response, and thus further investigation into the role of endocannabinoids in regulating thyroid activity during NTIS is warranted.

In the present study, expression of *CNR2* was most abundant in the SPN, which is consistent with previous work showing that this receptor is predominantly found in lymphoid tissues [26]. In fact, CNR2 was originally identified in the rat SPN where it localized to the marginal zones around the periarteriolar lymphoid sheaths, with expression confirmed in isolated splenic macrophages [38]. Expression on CNR2 has subsequently been demonstrated in both splenic and bone marrow B-cells, where is appears to play a role in retention [39, 40]. Steady state expression of both *CNR1* and *CNR2* in liver has generally been found to be low or below the level of detection in other species [33, 41], which was also the case in the bovine liver in the current study.

An interesting and novel finding in the present study was the detection of *CNR2* expression in the bovine ruminal papillae. This receptor has been found in various regions of the gastrointestinal tract in rats [42] and guinea pigs [43]. Its expression has been associated with mucosal inflammation and it has therefore been a target for developing therapies for gastrointestinal disease such as inflammatory bowel diseases [44, 45]. The role of *CNR2* in the ruminal papillae is not entirely clear. However, further analysis of CB2 protein expression using immunohistofluorescence staining revealed localization specifically in the ruminal epithelial cells. We have previously shown that the ruminal epithelium produces a local immune response following subacute ruminal acidosis, which is induced in ruminants following rapid dietary transitions [46]. More critically we have demonstrated that this immune response within the ruminal epithelial cells can be modulated by cannabidiol [47]. While CBD is not generally considered to be a ligand for CB2 in mice or humans, splicing of this gene appears to be species specific [48], and may influence receptor affinity. In addition, CBD has been shown to upregulate expression of CB2 [49], which may increase sensitivity to the immune suppressive effects of this receptor in response to other ligands. Beyond the immune system, other studies have shown a clear links between the ECS and metabolic regulation [50], although most implicate CB1 [51]. Studies have additionally provided evidence for a role of the CB2 receptor in nutrient absorption and metabolism [52]. For example, BCP, a full CB2 agonist, was found to have inhibitory activity on a-glucosidase, suggesting an ability to influence carbohydrate absorption in the small intestine [53]. Given the critical function of nutrient absorption and metabolism by the ruminal epithelium, fully elucidating the roles of CB2 in this unique tissue would provide insight into the potential utility and benefits of the ECS in the ruminant gastrointestinal tract.

An examination of the remaining bovine gastrointestinal tissues showed a near complete absence of the target receptors above baseline. These results initially appear to be counter the literature, which has demonstrated both CB1 and CB2 activity throughout the gastrointestinal tract in multiple species [44, 45]. However, much of the focus has been on evaluating the ECS during perturbations, such as gastrointestinal inflammation [44]. As is the case with many regulatory receptors, it is likely that the *CNR1* and *CNR2* gene expression are dependent on activation [54]. A limitation of the current study was there was no treatment included that might have induced a response by the ECS; the results of the current study are reflective of baseline levels in objectively healthy and physiologically stable steers. As such, further investigation into the gastrointestinal expression of these receptors following various physiological or pathological stressors is required.

Endocannabinoid levels are determined by a complex coordination of the synthesis and degradation enzymes [55, 56]. Synthesis and release occurs on demand in response to various stimuli, such as an increase in postsynaptic intracellular calcium [55] or activation of M1 and M3 receptors [57]. In the present study, transcript abundance for genes associated with endocannabinoid synthesis, *NAPEPLD*, responsible for AEA synthesis, and *DAGLA* and *DAGLB*, for 2-AG synthesis, was highly variable, both between the different tissues and animals. High levels of expression were detected in the BRN for all three of the enzymes. Gastrointestinal expression of *NAPEPLD*, critical for AEA synthesis, was greater in the small intestinal tissues and COL compared to the tissues of the upper gastrointestinal tract. Synthesis of 2-AG relies on two diacylglycerol lipases, DAGLA and DAGLB, the latter of which differs by the lack of a C-terminal tail [56]. While DAGLA is largely considered the primary synthesis enzyme for 2-AG in the CNS [58], previous work has provided evidence that expression of these two enzymes is highly tissue-dependent in other species, such as mice [56]. Interestingly, *DAGLA* was not found to be expressed in liver, tongue or rumen, and at relatively low levels in all other tissues except brain. Expression of *DAGLB*, however, was detectable in all tissues, but was highly variable between samples. Localization of DAGLB was further explored in the gastrointestinal tissues. In the tongue, positive staining was found in the cells of the circumvallate papillae. This is consistent with previous work showing expression of DAGLA and detection of 2-AG in mouse taste cells [59]. Moreover, the ECS has been implicated in regulating taste buds, especially in the modulation of “sweet” taste [60]. DAGLB was also found localized to epithelial cells of the rumen, small intestine and colon in the present study. In tissues of the middle and lower gastrointestinal tract, DAGLB appeared to stain goblet cells in the jejunum and ileum. In the COL, staining of goblet cells and enterocytes was observed. Similarly, Marquez et al. [61] demonstrated localization of both DAGLA and DAGLB in various epithelial cell types, including epithelial glands, in the human colon, and expression is altered by epithelial perturbations from ulcerative colitis. In the bovine gastrointestinal tract the endocannabinoid synthesis likely plays comparable roles in mediating inflammation in the epithelium.

Previous studies have demonstrated important roles for AEA and 2-AG in regulating intake, gut motility and energy balance [51, 62, 63]. A study by Gomez et al. [64] showed substantial increases in small intestinal AEA of starved rats. Although AEA levels were not measured in the present study, the high degree of *NAPEPLD* expression in the lower gastrointestinal tract may be attributed to their postprandial status, or due to stress of transport on the day of slaughter. Consistent with this theory, Kuhla et al. [14] found elevated plasma levels of both AEA and 2-AG in early lactation dairy cows and administration of both ECs improved feed intake during a stress response in late lactation Simmentals [15], suggesting possible orexigenic effects. In contrast, however, dry matter intake was not altered when AEA was administered to early lactation dairy cows [13].

In addition to synthesis enzymes, the balance of endocannabinoid activity relies on two key degradation enzymes, FAAH and MGLL. While FAAH is responsible for the metabolism of AEA, MGLL primarily degrades 2-AG [65]. In the present experiment, gene expression of *FAAH* was only detectable in a subset of the tissues examined. Among the gut tissues, only those of the small intestine (MJE and ILE) showed any meaningful expression. The action of AEA is tightly regulated through its degradation pathways and has a very short half-life [66, 67]. Therefore, expression may not be detectable depending on the physiological status if the tissue [23]. In contrast, *MGLL* was expressed, at various levels, in all tissues. 2-AG is an important source of arachidonic acid, a critical component of phospholipid membranes, with many physiological roles [56]. Expression levels in the various tissues may reflect mechanisms for maintaining physiological levels of arachidonic acid [58]. Moreover, MGLL has also been implicated in degradation of other monoacylglycerols besides 2-AG, suggesting its expression may be related to different functions, depending on the tissue type [65]. Overall the degree of expression of the degradation enzymes in the current study, in combination with the synthesis enzymes, likely contribute to maintaining basal concentrations of endocannabinoids and is reflective of the dynamic nature of the ECS in the bovine.

In addition to components of the ECS, this study also investigated a related system, the transient receptor potential (TRPs) channels. The TRPs are a class of membrane-bound, non-selective, cation channels, which are involved in various sensory functions, such as temperature and pain perception [68]. The TRP system is intertwined with the ECS and there may be many beneficial targets for developing therapies, for example to treat pain and inflammation [69]. The primary focus for research has been on the interactions between ECs and the TRPV1 receptor. There are many other TRPs proposed to interact with the ECS, including TRPV3 [70] and TRPV6 [71], however most have received little attention.

Expression of *TRPV3* has been detected in bovine rumen, and is proposed as a mediator of calcium and NH4 + absorption [72, 73]. Consistent with these previous findings, the present study also found expression of TRPV3 in the RUM. Expression was additionally detected in TNG, OMA and COL. Previous work has demonstrated TRPV3 localization in human tongue epithelium [74] and in mouse primary colonocytes [75]. Expression of *TRPV3* in bovine omasum has not been previously investigated. *TRPV6* was highly expressed in the THY and in the DUO tissue, however it was not at detectable levels in any of the other tissues of the gastrointestinal tract. While *TRPV6* was below the limit of detection in bovine RUM in Rosendahl et al. [76], expression was detected in small intestinal tissues, and only a low levels in the rumen in sheep [77]. Duodenal *TRPV6* also detected in bovine tissue [78], and expression has been found to be influenced by 1,25(OH)2D3 concentrations in humans [79]. Evidence suggests that phytochemicals such as terpenes and cannabinoids may interact with TRPV3 and TRPV6, and influence cation transport in the gastrointestinal tract. For example, menthol is a proposed agonist for TRPV3 and was thought to alter calcium transport in the ovine rumen [80]. Neuberger et al. [71] provided evidence of TRPV6 inhibition by the minor cannabinoid tetrahydrocannabivarin. Inhibitors of TRPV6 have gained recent interest for their potential use as therapies for various diseases relating to calcium dysregulation, including in the gastrointestinal tract and the thyroid gland [81, 82].

Although the focus of this study was on the transcript abundance of the ECS across various tissues, an interesting aspect that might have added some additional insight is quantified protein expression. Moreover, a limitation of the expression data of the intestinal tissues is a lack of specificity with regards to cell type, due to the use of whole mucosa. Exploration of the expression in various cell types would further the knowledge of the ECS, however this analysis was beyond the scope of the current study.

## Conclusions

While the knowledge of this system in ruminants is not extensive, evidence suggests there are many opportunities for utilizing the ECS to benefit production animals [83]. Phytochemicals that interact with the various components of the system may be useful for mitigating pain or stress [11, 69], or for altering intake and lipid metabolism [12, 15]. An understanding of the constitutive transcript abundance will aid in the identification of tissue-specific targets. In conclusion, this experiment demonstrates the dynamic nature of expression of the bovine ECS. The results provide novel insights into the steady-state expression of the ECS in bovine tissue, expanding the understanding of the system and providing a stepping off point for future investigations into the potential benefits for ruminants.

## Electronic supplementary material

Below is the link to the electronic supplementary material.

Supplementary Material 1

## Data Availability

The data supporting this study’s findings are in the manuscript and supplementary file and are also available from the corresponding author upon reasonable request.

## References

[1] Sharkey KA, Wiley JW. The role of the endocannabinoid system in the brain–gut axis. Gastroenterology. 2016;151:252–66. 27133395 10.1053/j.gastro.2016.04.015 PMC4961581

[2] Matsuda LA, Lolait SJ, Brownstein MJ, Young AC, Bonner TI. Structure of a cannabinoid receptor and functional expression of the cloned cDNA. Nature. 1990;346:561–4. 2165569 10.1038/346561a0

[3] Silver RJ. The endocannabinoid system of animals. Animals. 2019;9:686. 31527410 10.3390/ani9090686 PMC6770351

[4] Ligresti A, De Petrocellis L, Di Marzo V. From phytocannabinoids to cannabinoid receptors and endocannabinoids: pleiotropic physiological and pathological roles through complex pharmacology. Physiol Rev. 2016;96:1593–659. 27630175 10.1152/physrev.00002.2016

[5] Jamontt J, Molleman A, Pertwee R, Parsons M. The effects of ∆9-tetrahydrocannabinol and cannabidiol alone and in combination on damage, inflammation and in vitro motility disturbances in rat colitis. Br J Pharmacol. 2010;160:712–23. 20590574 10.1111/j.1476-5381.2010.00791.x PMC2931570

[6] Ligresti A, Villano R, Allarà M, Ujváry I, Di Marzo V. Kavalactones and the endocannabinoid system: the plant-derived yangonin is a novel CB1 receptor ligand. Pharmacol Res. 2012;66:163–9. 22525682 10.1016/j.phrs.2012.04.003

[7] Gertsch J, Leonti M, Raduner S, Racz I, Chen J-Z, Xie X-Q et al. Beta-caryophyllene is a dietary cannabinoid. Proceedings of the National Academy of Sciences. 2008;105:9099–104. 10.1073/pnas.0803601105 PMC2449371 18574142

[8] Bento AF, Marcon R, Dutra RC, Claudino RF, Cola M, Leite DFP, et al. β-Caryophyllene inhibits Dextran Sulfate Sodium-Induced Colitis in mice through CB2 receptor activation and PPARγ pathway. Am J Pathol. 2011;178:1153–66. 21356367 10.1016/j.ajpath.2010.11.052 PMC3070571

[9] Basha RH, Sankaranarayanan C. β-Caryophyllene, a natural sesquiterpene lactone attenuates hyperglycemia mediated oxidative and inflammatory stress in experimental diabetic rats. Chemico-Biol Interact. 2016;245:50–8. 10.1016/j.cbi.2015.12.019 26748309

[10] Scarpelli R, Sasso O, Piomelli D. A double whammy: targeting both fatty acid amide hydrolase (FAAH) and cyclooxygenase (COX) to treat pain and inflammation. ChemMedChem. 2015;11:1242–51. 26486424 10.1002/cmdc.201500395 PMC4840092

[11] Muller C, Morales P, Reggio PH. Cannabinoid ligands targeting TRP channels. Front Mol Neurosci. 2019;11. 10.3389/fnmol.2018.00487 PMC6340993 30697147

[12] Myers MN, Abou-Rjeileh U, Chirivi M, Parales-Girón J, Lock AL, Tam J, et al. Cannabinoid-1 receptor activation modulates lipid mobilization and adipogenesis in the adipose tissue of dairy cows. J Dairy Sci. 2023;106:3650–61. 36907764 10.3168/jds.2022-22556

[13] Schwerdtfeger J, Sauerwein H, Albrecht E, Mazzuoli-Weber G, Von Soosten D, Dänicke S et al. The effect of N-arachidonoylethanolamide administration on energy and fat metabolism of early lactating dairy cows. Sci Rep. 2023;13. 10.1038/s41598-023-41938-0 PMC10482912 37673919

[14] Kuhla B, Kaever V, Tuchscherer A, Kuhla A. Involvement of plasma endocannabinoids and the Hypothalamic Endocannabinoid System in increasing feed intake after parturition of dairy cows. Neuroendocrinology. 2019;110:246–57. 31141804 10.1159/000501208

[15] Van Ackern I, Wulf R, Dannenberger D, Tuchscherer A, Kuhla B. Effects of endocannabinoids on feed intake, stress response and whole-body energy metabolism in dairy cows. Sci Rep. 2021;11. 10.1038/s41598-021-02970-0 PMC8655048 34880316

[16] Oh J, Wall EH, Bravo DM, Hristov AN. Host-mediated effects of phytonutrients in ruminants: a review. J Dairy Sci. 2017;100:5974–83. 28390713 10.3168/jds.2016-12341

[17] Trotta RJ, Swanson KC, Klotz JL, Harmon DL. Postruminal casein infusion and exogenous Glucagon-Like peptide 2 administration differentially stimulate pancreatic Α-Amylase and small intestinal Α-Glucosidase activity in cattle. J Nutr. 2023;153:2854–67. 37573014 10.1016/j.tjnut.2023.08.009

[18] Klotz JL, Barnes AJ. Isolating and using sections of bovine mesenteric artery and vein as a bioassay to test for vasoactivity in the small intestine. J Visualized Experiments. 2014. 10.3791/52020. 10.3791/52020 PMC4841295 25350042

[19] Ison EK, Hopf-Jannasch AS, Harding JCS, Pasternak JA. Effects of porcine reproductive and respiratory syndrome virus (PRRSV) on thyroid hormone metabolism in the late gestation fetus. Vet Res. 2022;53. 10.1186/s13567-022-01092-3 PMC9524047 36175938

[20] Team RC. (2018) R: A language and environment for statistical computing. Vienna: Austria; 2018.

[21] Pfaffl MW. A new mathematical model for relative quantification in real-time RT-PCR. Nucleic Acids Res. 2001;29:e45–45. 11328886 10.1093/nar/29.9.e45 PMC55695

[22] Wickham H. ggplot2: elegant graphics for data analysis. New York: Springer; 2016.

[23] Laprairie R, Kelly M, Denovan-Wright E. The dynamic nature of type 1 cannabinoid receptor (CB1) gene transcription. Br J Pharmacol. 2012;167:1583–95. 22924606 10.1111/j.1476-5381.2012.02175.x PMC3525862

[24] Busquets-Garcia A, Bains J, Marsicano G. CB1 receptor signaling in the brain: extracting specificity from Ubiquity. Neuropsychopharmacology. 2017;43:4–20. 28862250 10.1038/npp.2017.206 PMC5719111

[25] Garcia AB, Soria-Gomez E, Bellocchio L, Marsicano G. Cannabinoid receptor type-1: breaking the dogmas. F1000Research. 2016;5:990. 10.12688/f1000research.8245.1 PMC4879932 27239293

[26] Hasenoehrl C, Taschler U, Storr M, Schicho R. The gastrointestinal tract – a central organ of cannabinoid signaling in health and disease. Neurogastroenterology Motil. 2016;28:1765–80. 10.1111/nmo.12931 PMC5130148 27561826

[27] Elphick MR, Egertova M. The neurobiology and evolution of cannabinoid signalling. Philosophical Trans Royal Soc B Biol Sci. 2001;356:381–408. 10.1098/rstb.2000.0787 PMC1088434 11316486

[28] Herkenham M, Lynn A, Johnson, Melvin L, De Costa B, Rice K. Characterization and localization of cannabinoid receptors in rat brain: a quantitative in vitro autoradiographic study. J Neurosci. 1991;11:563–83. 1992016 10.1523/JNEUROSCI.11-02-00563.1991 PMC6575215

[29] Kendall DA, Yudowski GA. Cannabinoid receptors in the central nervous system: their signaling and roles in disease. Front Cell Neurosci. 2017;10. 10.3389/fncel.2016.00294 PMC5209363 28101004

[30] Martin AMS, Kim D-J, Newman SD, Cheng H, Hetrick WP, Mackie K, et al. Altered cerebellar-cortical resting-state functional connectivity in cannabis users. J Psychopharmacol. 2021;35:823–32. 34034553 10.1177/02698811211019291 PMC8813046

[31] Wagner B, Gerletti P, Fürst P, Keuth O, Bernsmann T, Martin A, et al. Transfer of cannabinoids into the milk of dairy cows fed with industrial hemp could lead to ∆9-THC exposure that exceeds acute reference dose. Nat Food. 2022;3:921–32. 37118216 10.1038/s43016-022-00623-7

[32] Lakiotaki E, Giaginis C, Tolia M, Alexandrou P, Delladetsima I, Giannopoulou I, et al. Clinical significance of cannabinoid receptors CB1 and CB2 expression in human malignant and benign thyroid lesions. Biomed Res Int. 2015;2015:1–7. 10.1155/2015/839403 PMC4619873 26539529

[33] Porcella A, Marchese G, Casu MA, Rocchitta A, Lai ML, Gessa GL, et al. Evidence for functional CB1 cannabinoid receptor expressed in the rat thyroid. Eur J Endocrinol. 2002;147:255–61. 12153749 10.1530/eje.0.1470255

[34] Hillard CJ. Endocannabinoids and the endocrine system in health and disease. Handb Exp Pharmacol. 2015;231:317–39. 10.1007/978-3-319-20825-1_11 PMC6813821 26408166

[35] Vassiliadi DA, Ilias I, Pratikaki M, Jahaj E, Vassiliou AG, Detsika M, et al. Thyroid hormone alterations in critically and non-critically ill patients with SARS-CoV-2 infection. Endocr Connections. 2021;10:646–55. 10.1530/EC-21-0029 PMC8240704 34010152

[36] Pasternak JA, MacPhee DJ, Lunney JK, Rowland RRR, Dyck MK, Fortin F et al. Thyroid hormone suppression in feeder pigs following polymicrobial or porcine reproductive and respiratory syndrome virus-2 challenge. J Anim Sci. 2021;99. 10.1093/jas/skab325 PMC8633620 34734242

[37] Pasternak JA, MacPhee DJ, Harding JCS. Maternal and fetal thyroid dysfunction following porcine reproductive and respiratory syndrome virus2 infection. Vet Res. 2020;51. 10.1186/s13567-020-00772-2 PMC7106657 32228691

[38] Munro S, Thomas KL, Abu-Shaar M. Molecular characterization of a peripheral receptor for cannabinoids. Nature. 1993;365:61–5. 7689702 10.1038/365061a0

[39] Pereira JP, An J, Xu Y, Huang Y, Cyster JG. Cannabinoid receptor 2 mediates the retention of immature B cells in bone marrow sinusoids. Nat Immunol. 2009;10:403–11. 19252491 10.1038/ni.1710 PMC2768754

[40] Muppidi JR, Arnon TI, Bronevetsky Y, Veerapen N, Tanaka M, Besra GS, et al. Cannabinoid receptor 2 positions and retains marginal zone B cells within the splenic marginal zone. J Exp Med. 2011;208:1941–8. 21875957 10.1084/jem.20111083 PMC3182059

[41] Kim Y, Gautam S, Aseer KR, Kim J, Chandrasekaran P, Mazucanti CH et al. Hepatocyte cannabinoid 1 receptor nullification alleviates toxin-induced liver damage via NF-κB signaling. Cell Death Dis. 2020;11. 10.1038/s41419-020-03261-8 PMC7726564 33298885

[42] Storr M, Gaffal E, Saur D, Schusdziarra V, Allescher HD. Effect of cannabinoids on neural transmission in rat gastric fundus. Can J Physiol Pharmacol. 2002;80:67–76. 11911227 10.1139/y02-005

[43] Griffin G, Fernando SR, Ross RA, McKay NG, Ashford MLJ, Shire D, et al. Evidence for the presence of CB2-like cannabinoid receptors on peripheral nerve terminals. Eur J Pharmacol. 1997;339:53–61. 9450616 10.1016/s0014-2999(97)01336-8

[44] Wright KL, Duncan M, Sharkey KA. Cannabinoid CB2 receptors in the gastrointestinal tract: a regulatory system in states of inflammation. Br J Pharmacol. 2008;153:263–70. 17906675 10.1038/sj.bjp.0707486 PMC2219529

[45] Scheau C, Caruntu C, Badarau IA, Scheau A-E, Docea AO, Calina D, et al. Cannabinoids and inflammations of the Gut-Lung-Skin barrier. J Personalized Med. 2021;11:494. 10.3390/jpm11060494 PMC8227007 34072930

[46] Kent-Dennis C, Pasternak A, Plaizier JC, Penner GB. Potential for a localized immune response by the ruminal epithelium in nonpregnant heifers following a short-term subacute ruminal acidosis challenge. J Dairy Sci. 2019;102:7556–69. 31229286 10.3168/jds.2019-16294

[47] Kent-Dennis C, Klotz JL. Immunomodulation by cannabidiol in bovine primary ruminal epithelial cells. BMC Vet Res. 2023;19. 10.1186/s12917-023-03756-4 PMC10577946 37845710

[48] Liu Q-R, Pan C-H, Hishimoto A, Li C-Y, Xi Z-X, Llorente-Berzal A, et al. Species differences in cannabinoid receptor 2 (CNR2 gene): identification of novel human and rodent CB2 isoforms, differential tissue expression and regulation by cannabinoid receptor ligands. Genes Brain Behav. 2009;8:519–30. 19496827 10.1111/j.1601-183X.2009.00498.x PMC3389515

[49] Gasparyan A, Navarrete F, Rodríguez-Arias M, Miñarro J, Manzanares J. Cannabidiol modulates behavioural and gene expression alterations induced by spontaneous cocaine withdrawal. Neurotherapeutics. 2021;18:615–23. 33230690 10.1007/s13311-020-00976-6 PMC8116402

[50] Silvestri C, Di Marzo V. The endocannabinoid system in energy homeostasis and the etiopathology of metabolic disorders. Cell Metabol. 2013;17:475–90. 10.1016/j.cmet.2013.03.001 23562074

[51] Quarta C, Mazza R, Obici S, Pasquali R, Pagotto U. Energy balance regulation by endocannabinoids at central and peripheral levels. Trends Mol Med. 2011;17:518–26. 21816675 10.1016/j.molmed.2011.05.002

[52] Hashiesh HM, Meeran MFN, Sharma C, Sadek B, Kaabi JA, Ojha SK. Therapeutic potential of Β-Caryophyllene: a dietary cannabinoid in diabetes and associated complications. Nutrients. 2020;12:2963. 32998300 10.3390/nu12102963 PMC7599522

[53] Kaur G, Tharappel LJP, Kumawat V. Evaluation of Safety and in vitro mechanisms of anti-diabetic activity of β-caryophyllene and L-arginine. J Biol Sci. 2018;18:124–34.

[54] Fournier T, Gabriel JP, Mazza C, Pasquier J, Galbete JL, Mermod N. Steady-state expression of self-regulated genes. Bioinformatics. 2007;23:3185–92. 17933850 10.1093/bioinformatics/btm490

[55] Alger BE, Kim J. Supply and demand for endocannabinoids. Trends Neurosci. 2011;34:304–15. 21507493 10.1016/j.tins.2011.03.003 PMC3106144

[56] Baggelaar MP, Maccarrone M, Van Der Stelt M. 2-Arachidonoylglycerol: a signaling lipid with manifold actions in the brain. Prog Lipid Res. 2018;71:1–17. 29751000 10.1016/j.plipres.2018.05.002

[57] Ohno-Shosaku T, Matsui M, Fukudome Y, Shosaku J, Tsubokawa H, Taketo MM, et al. Postsynaptic M1 and M3 receptors are responsible for the muscarinic enhancement of retrograde endocannabinoid signalling in the hippocampus. Eur J Neurosci. 2003;18:109–16. 12859343 10.1046/j.1460-9568.2003.02732.x

[58] Gao Y, Vasilyev DV, Goncalves MB, Howell FV, Hobbs C, Reisenberg M, et al. Loss of retrograde endocannabinoid signaling and reduced adult neurogenesis in diacylglycerol lipase knock-out mice. J Neurosci. 2010;30:2017–24. 20147530 10.1523/JNEUROSCI.5693-09.2010 PMC6634037

[59] Niki M, Jyotaki M, Yoshida R, Yasumatsu K, Shigemura N, DiPatrizio NV, et al. Modulation of sweet taste sensitivities by endogenous leptin and endocannabinoids in mice. J Physiol. 2015;593:2527–45. 25728242 10.1113/JP270295 PMC4461413

[60] Tarragon E, Moreno JJ. Cannabinoids, chemical senses, and regulation of feeding behavior. Chem Senses. 2018;44:73–89. 10.1093/chemse/bjy068 30481264

[61] Marquéz L, Suárez J, Iglesias M, Bermudez-Silva FJ, De Fonseca FR, Andreu M. Ulcerative colitis induces changes on the expression of the endocannabinoid system in the human colonic tissue. PLoS ONE. 2009;4:e6893. 19730730 10.1371/journal.pone.0006893 PMC2731878

[62] Capasso R, Izzo AA. Gastrointestinal regulation of food intake: general aspects and focus on anandamide and oleoylethanolamide. J Neuroendocrinol. 2008;20:39–46. 18426498 10.1111/j.1365-2826.2008.01686.x

[63] Mock ED, Gagestein B, Van Der Stelt M. Anandamide and other N-acylethanolamines: a class of signaling lipids with therapeutic opportunities. Prog Lipid Res. 2023;89:101194. 36150527 10.1016/j.plipres.2022.101194

[64] Gómez R, Navarro M, Ferrer B, Trigo JM, Bilbao A, Del Arco I, et al. A peripheral mechanism for CB1 Cannabinoid receptor-dependent modulation of feeding. J Neurosci. 2002;22:9612–7. 12417686 10.1523/JNEUROSCI.22-21-09612.2002 PMC6758016

[65] Tsuboi K, Uyama T, Okamoto Y, Ueda N. Endocannabinoids and related N-acylethanolamines: biological activities and metabolism. Inflamm Regeneration. 2018;38. 10.1186/s41232-018-0086-5 PMC6166290 30288203

[66] Cravatt BF, Demarest K, Patricelli MP, Bracey MH, Giang DK, Martin BR, et al. Supersensitivity to anandamide and enhanced endogenous cannabinoid signaling in mice lacking fatty acid amide hydrolase. Proc Natl Acad Sci. 2001;98:9371–6. 11470906 10.1073/pnas.161191698 PMC55427

[67] Atkinson DL, Abbott JK. Cannabinoids and the brain: The effects of endogenous and exogenous cannabinoids on brain systems and function. In: Elsevier eBooks. 2018. pp. 37–74.

[68] Zhang M, Ma Y, Ye X, Zhang N, Pan L, Wang B. TRP (transient receptor potential) ion channel family: structures, biological functions and therapeutic interventions for diseases. Signal Transduct Target Therapy. 2023;8. 10.1038/s41392-023-01464-x PMC10319900 37402746

[69] De Petrocellis L, Ligresti A, Moriello AS, Allarà M, Bisogno T, Petrosino S, et al. Effects of cannabinoids and cannabinoid-enriched Cannabis extracts on TRP channels and endocannabinoid metabolic enzymes. Br J Pharmacol. 2011;163:1479–94. 21175579 10.1111/j.1476-5381.2010.01166.x PMC3165957

[70] Storozhuk MV, Zholos AV. TRP channels as novel targets for endogenous ligands: focus on endocannabinoids and nociceptive signalling. Curr Neuropharmacol. 2018;16. 10.2174/1570159X15666170424120802 PMC5883376 28440188

[71] Neuberger A, Trofimov YA, Yelshanskaya MV, Khau J, Nadezhdin KD, Khosrof LS et al. Molecular pathway and structural mechanism of human oncochannel TRPV6 inhibition by the phytocannabinoid tetrahydrocannabivarin. Nat Commun. 2023;14. 10.1038/s41467-023-40362-2 PMC10397291 37532722

[72] Liebe F, Liebe H, Kaessmeyer S, Sponder G, Stumpff F. The TRPV3 channel of the bovine rumen: localization and functional characterization of a protein relevant for ruminal ammonia transport. Pflügers Archiv - Eur J Physiol. 2020;472:693–710. 32458085 10.1007/s00424-020-02393-2 PMC7293678

[73] Liebe F, Liebe H, Sponder G, Mergler S, Stumpff F. Effects of butyrate – on ruminal Ca2 + transport: evidence for the involvement of apically expressed TRPV3 and TRPV4 channels. Pflügers Archiv - Eur J Physiol. 2022;474:315–42. 35098357 10.1007/s00424-021-02647-7 PMC8837523

[74] Moayedi Y, Michlig S, Park M, Koch A, Lumpkin EA. Localization of TRP Channels in healthy oral mucosa from human donors. eNeuro. 2022;9:ENEURO0328–212022. 10.1523/ENEURO.0328-21.2022 PMC9797210 36635242

[75] Bischof M, Olthoff S, Glas C, Thorn-Seshold O, Schaefer M, Hill K. TRPV3 endogenously expressed in murine colonic epithelial cells is inhibited by the novel TRPV3 blocker 26E01. Cell Calcium. 2020;92:102310. 33161279 10.1016/j.ceca.2020.102310

[76] Rosendahl J, Braun HS, Schrapers KT, Martens H, Stumpff F. Evidence for the functional involvement of members of the TRP channel family in the uptake of na + and NH4 + by the ruminal epithelium. Pflügers Archiv - Eur J Physiol. 2016;468:1333–52. 27184746 10.1007/s00424-016-1835-4

[77] Wilkens MR, Kunert-Keil C, Brinkmeier H, Schröder B. Expression of calcium channel TRPV6 in ovine epithelial tissue. Vet J. 2009;182:294–300. 18701326 10.1016/j.tvjl.2008.06.020

[78] Schröder B, Wilkens MR, Ricken GE, Leonhard-Marek S, Fraser DR, Breves G. Calcium transport in bovine rumen epithelium as affected by luminal ca concentrations and ca sources. Physiological Rep. 2015;3:e12615. 10.14814/phy2.12615 PMC4673643 26564067

[79] Balesaria S, Sangha S, Walters JRF. Human duodenum responses to vitamin D metabolites of TRPV6 and other genes involved in calcium absorption. AJP Gastrointest Liver Physiol. 2009;297:G1193–7. 10.1152/ajpgi.00237.2009 PMC2850091 19779013

[80] Geiger S, Patra AK, Schrapers KT, Braun HS, Aschenbach JR. Menthol stimulates calcium absorption in the rumen but not in the jejunum of sheep. J Dairy Sci. 2021;104:3067–81. 33358813 10.3168/jds.2020-19372

[81] Bhardwaj R, Lindinger S, Neuberger A, Nadezhdin KD, Singh AK, Cunha MR et al. Inactivation-mimicking block of the epithelial calcium channel TRPV6. Sci Adv. 2020;6. 10.1126/sciadv.abe1508 PMC7695471 33246965

[82] Stewart JM. TRPV6 as a target for cancer therapy. J Cancer. 2020;11:374–87. 31897233 10.7150/jca.31640 PMC6930427

[83] Altman A, Kent-Dennis C, Klotz J, McLeod K, Vanzant E, Harmon D. Review: utilizing industrial hemp (Cannabis sativa L.) by-products in livestock rations. Anim Feed Sci Technol. 2024;307:115850.

