# Supplementary material for: The endocannabinoid system in bovine tissues: characterization of transcript abundance in the growing Holstein steer
Source: BMC Vet Res. 2024 Oct 22;20:481. doi: 10.1186/s12917-024-04319-x (PMC11494806; doi:10.1186/s12917-024-04319-x)
Supplement: Supplementary file 1 — Supplementary Material 1 [file 12917_2024_4319_MOESM1_ESM.docx]

Supplementary Material: Immunohistofluorescence images show all separate channels and merged images for target antibodies, and corresponding isotype controls demonstrating minimal non-specific staining.


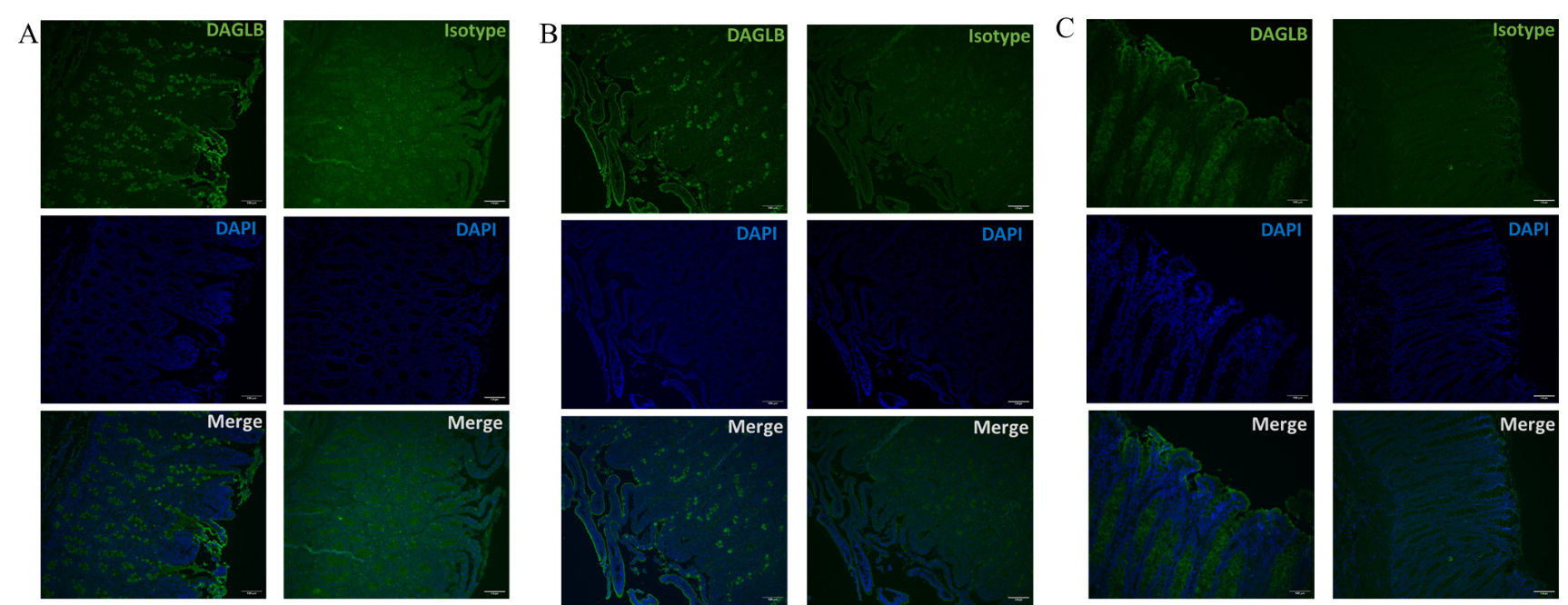

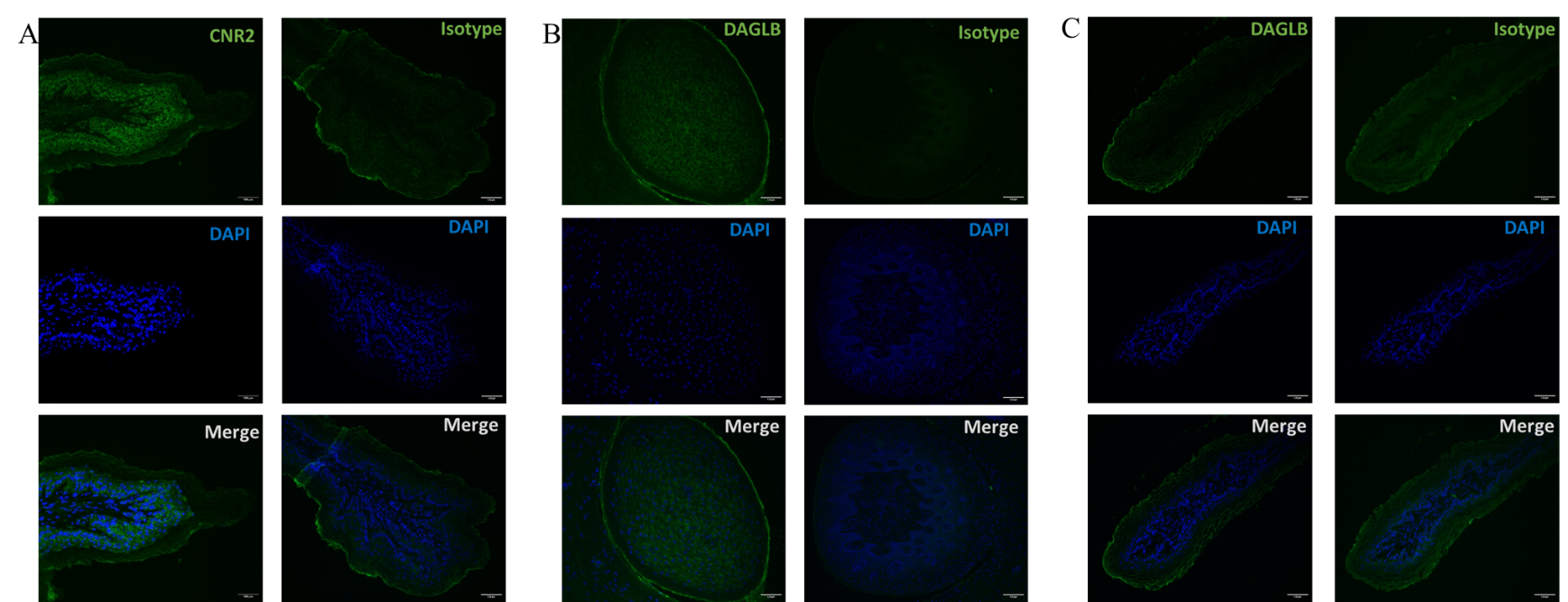


Supplementary Figure 2. Immunohistofluorescent staining of DAGLB and Isotype Control in duodenum (A), ileum (B) and colon (C). Nuclei are counterstained with DAPI (blue).

Supplementary Figure 1. Immunohistofluorescent staining of CNR2 and Isotype control in ruminal epithelium (A), and of DAGLB and Isotype Control in tongue (B) and ruminal epithelium (C). Nuclei are counterstained with DAPI (blue).
